# A Rare Case of Chronic Lunate Dislocation Along With Avascular Necrosis of the Lunate With Median Nerve Compression Treated With Lunate Excision With Carpal Tunnel Release

**DOI:** 10.7759/cureus.45957

**Published:** 2023-09-25

**Authors:** Sahil Chowdhary, Amit Kale

**Affiliations:** 1 Orthopaedics, Dr. D. Y. Patil Medical College, Hospital & Research Centre, Pune, IND

**Keywords:** avascular necrosis of lunate, proximal carpal arc, carpel tunnel release, lunate excision, median nerve injury, lunate dislocation

## Abstract

A young 22-year-old male presented with complaints of pain, tingling, and numbness over his right wrist for 1 year and had a history of falls on his outstretched hand. Radiological evaluations such as X-rays were done, which showed a break in the proximal carpal arc. An MRI of the affected wrist was done. MRI findings are suggestive of avascular necrosis of the lunate along with lunate dislocation with marrow edema/contusion in the lunate. Complete disruption of the scapholunate, lunotriquetral, and radioscaphocapitate ligaments was noted. The patient was operated with lunate excision with carpal tunnel release and given strict pillow cover elevation in a volar slab.

## Introduction

Lunate dislocations are injuries caused by high-energy mechanisms of injuries, such as road traffic accidents, falls from height, and sports injuries [1]. The classic mechanisms of injury are forceful wrist extension, ulnar deviation, and carpal supination [2]. According to the Mayfield classification, lunate dislocations are classified into stage IV injuries. Lunate dislocations are often associated with complete disruption of the scapholunate complex and dorsal radiocarpal ligament. This results in the lunate to rotate into the carpal tunnel [2]. Carpal tunnel syndrome (CTS) after an acute lunate dislocation has been reported in up to 50% of cases, and it is estimated that up to 25% of acute lunate dislocations are misdiagnosed initially [3]. These injuries are later identified because patients have chronic pain and symptoms of compressive neuropathy.

Patients with unreduced injuries may present very late (up to years) after the injury, although some of the cases may have good hand function with minimal pain [4]. We discuss a patient with chronic lunate dislocation along with avascular necrosis of the lunate who presented with pain and worsening signs and symptoms of CTS in his right wrist. He subsequently underwent lunate excision with carpal tunnel release. The gross anatomy at the time of operative treatment ultimately guided our surgical management.

## Case presentation

**Figure 1:**
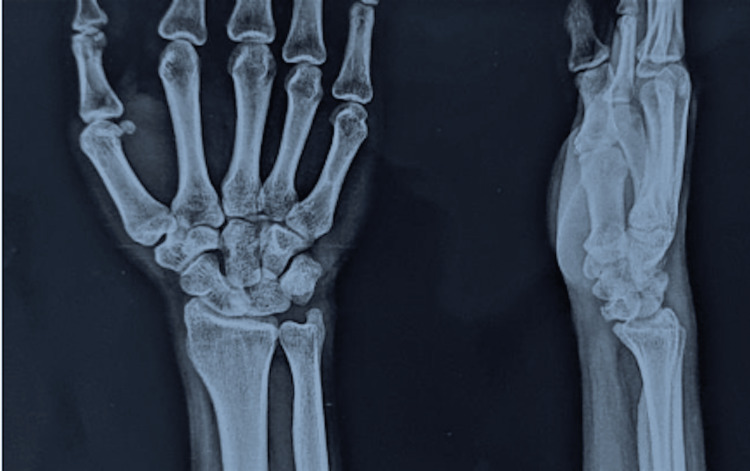
Preoperative X-ray showing a break in the proximal arc

**Figure 2:**
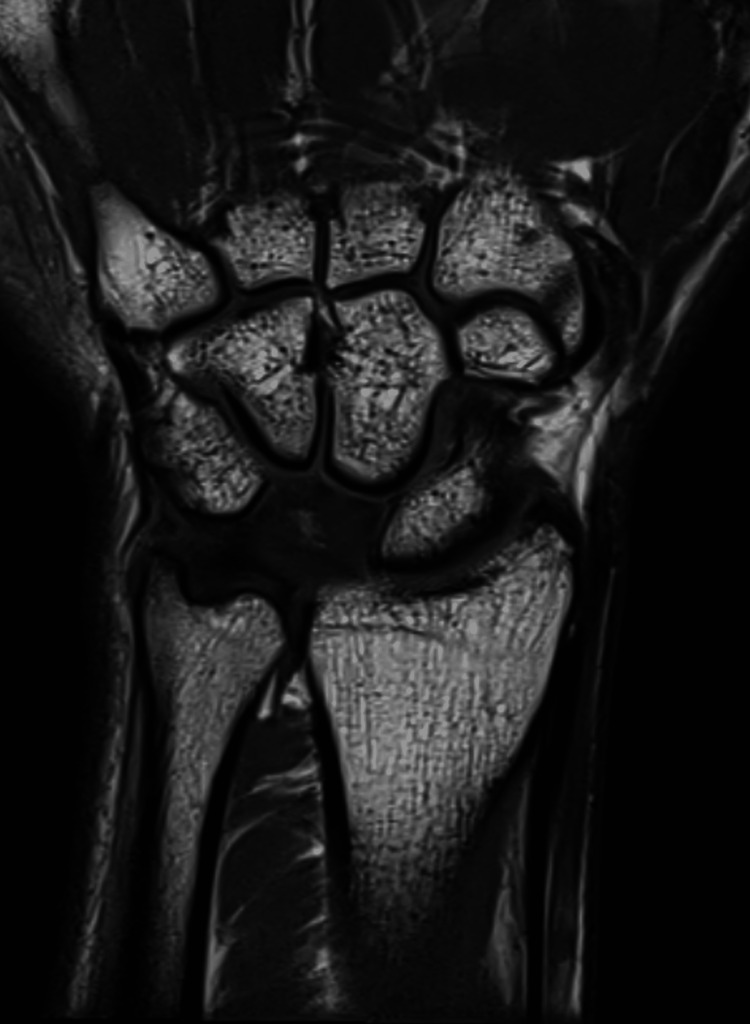
MRI showing avascular necrosis of the lunate

**Figure 3:**
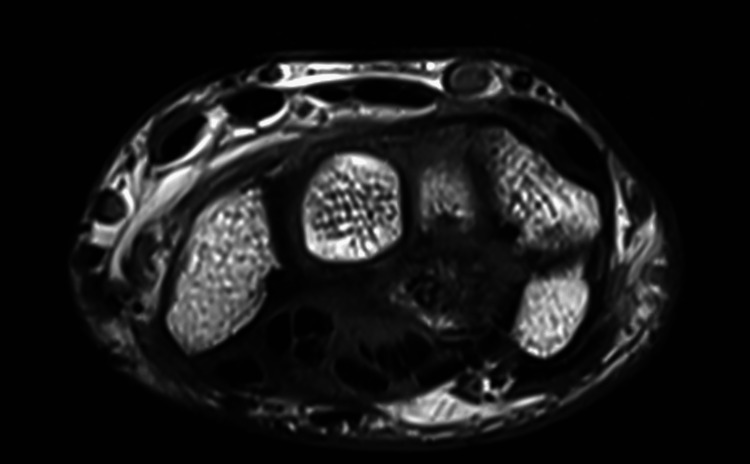
MRI showing the loss of normal smooth contour with cortical irregularity and marrow edema at the superior articular portion

**Figure 4:**
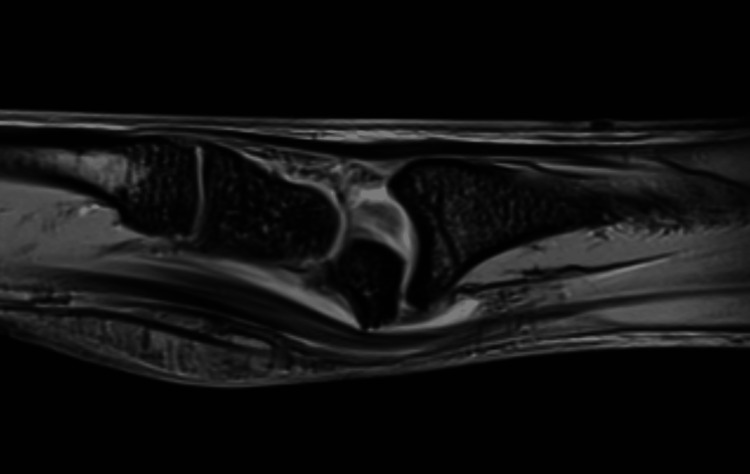
Linear incomplete fracture of the anterior superior portion of the lunate bone at the articular facet of the capitate

A young 22-year-old male presented with complaints of pain, tingling, and numbness over his right wrist for 1 year and had a history of falls on his outstretched hand. X-rays were done, which showed a break in the proximal carpal arc (Figure 1). An MRI of the affected wrist was done. MRI findings are suggestive of lunate dislocation, with complete disruption of the scapholunate, lunotriquetral, and radioscaphocapitate ligaments (Figures 2, 3, 4).

**Figure 5:**
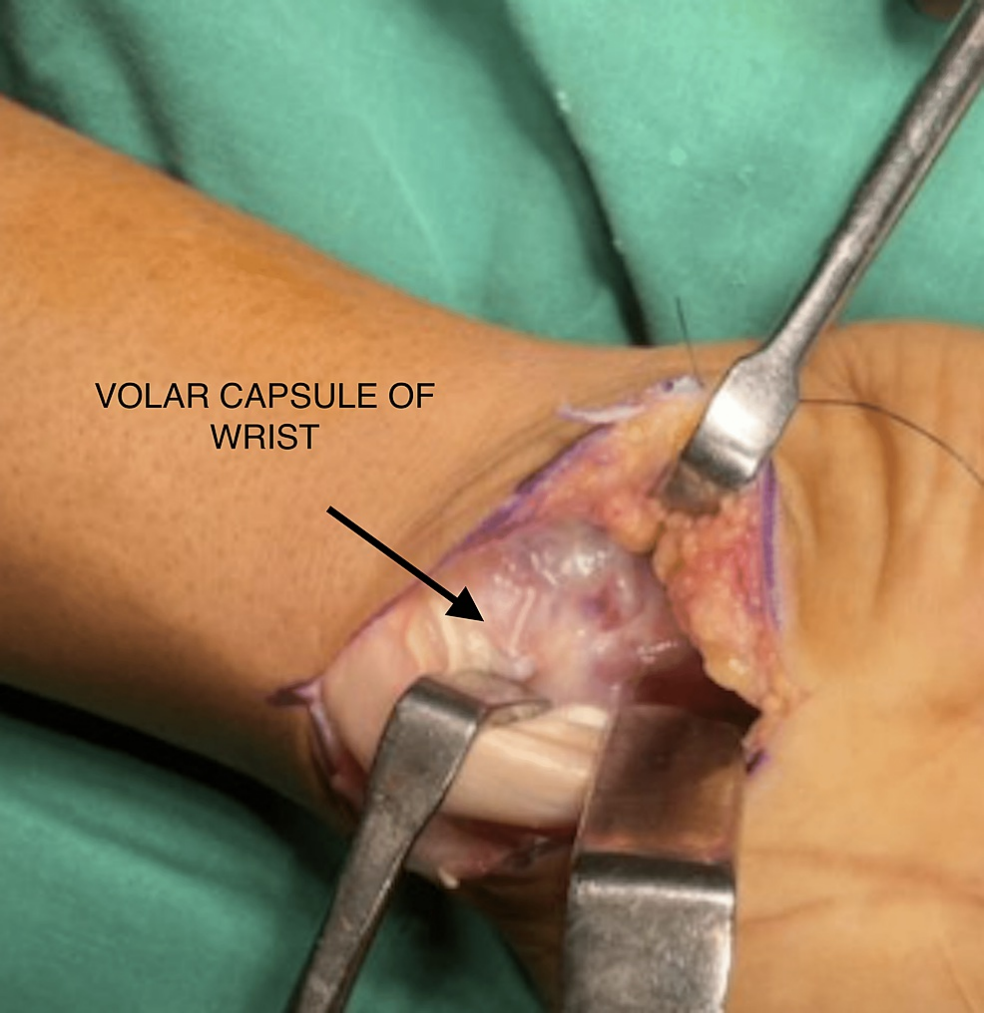
Volar incision of the wrist

**Figure 6:**
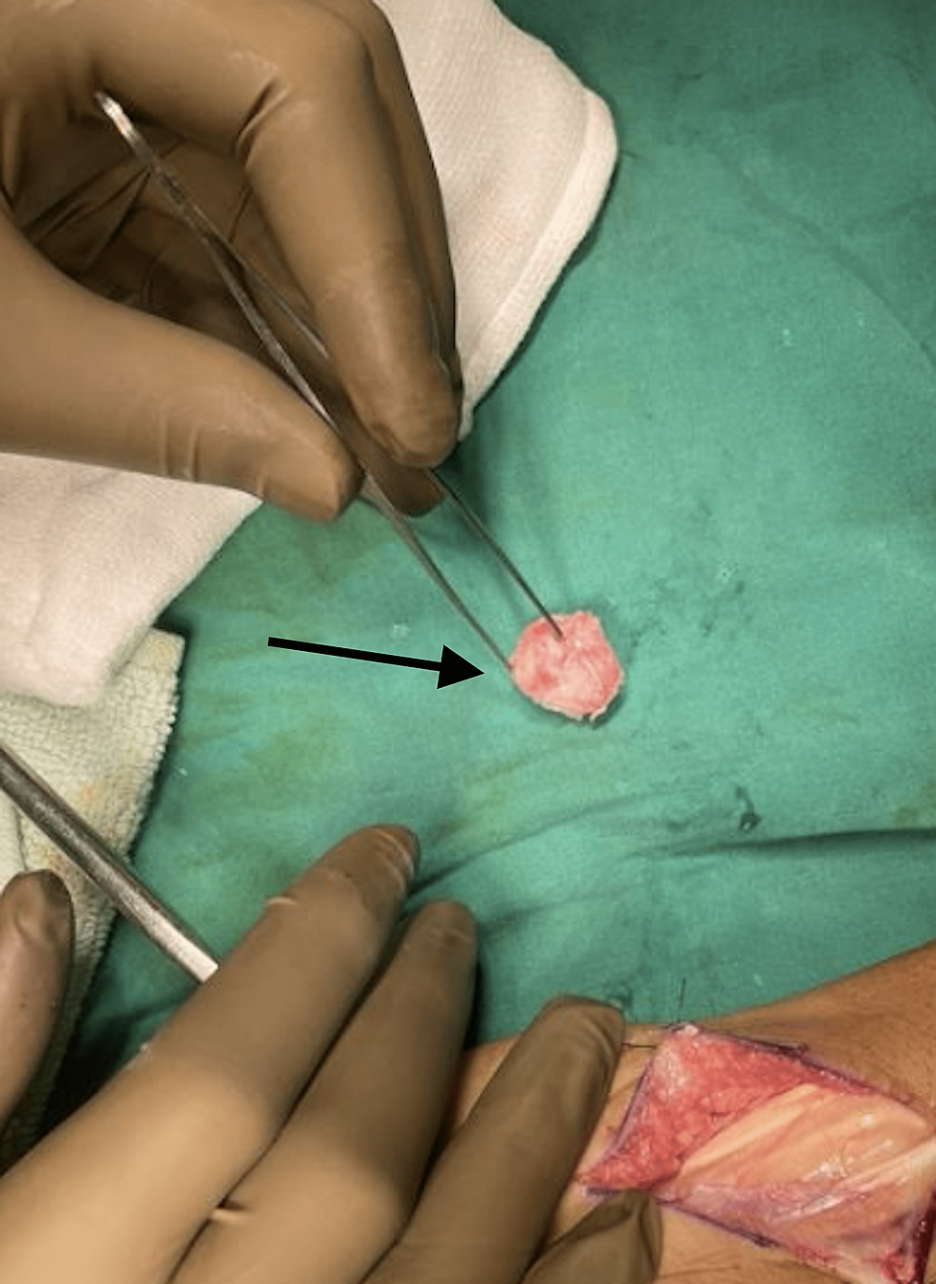
Intraoperative photo of the excised lunate from the right wrist

**Figure 7:**
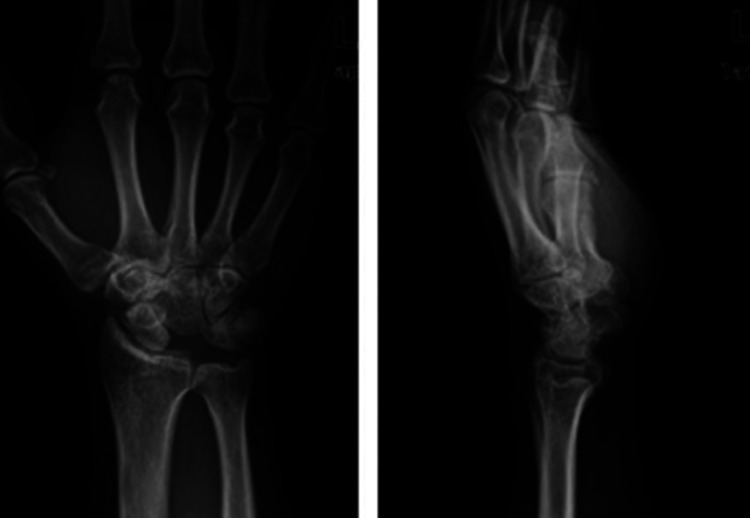
Postoperative X-ray after excision

The patient was taken for lunate excision with carpal tunnel release (Figure 5, 6, 7).

A volar slab with strict pillow cover elevation was given in the postoperative period for a better functional outcome. The patient was advised to follow up at regular intervals and postop rehabilitative physiotherapy under expert guidance.

## Discussion

CTS is the most common compressive neuropathy of the upper extremity, its incidence being 5-10% of the general population. The diagnosis is made clinically. The patient usually complains of a nighttime burning sensation and associated tingling/numbness in the thumb, middle, and index fingers. The most common cause of CTS is idiopathic. However local factors that contribute to CTS are inflammation, trauma, tumors, or anatomical abnormalities. Systemic factors include obesity, pregnancy, diabetes mellitus, rheumatoid arthritis, and osteoarthritis.

Symptoms arise due to median nerve compression that disrupts normal axonal transport and nerve function. In our patient, the acute fall likely resulted in lunate dislocation and compressing of the median nerve. This caused him to have features of compressive neuropathy, which coincided with the initial presentation. Several reports have been published on space-occupying lesions, such as lipoma [5] and ganglion [6].

Chronic lunate dislocations are rare injuries of the wrist. They usually result from high-energy trauma, leading to ligament disruption [7]. It is challenging to diagnose such dislocation; therefore, if such dislocation is left untreated, it may result in posttraumatic arthritis, chronic wrist pain, reduced grip strength, limited wrist range of motion, CTS, and attritional flexor tendon rupture [7].

Various treatment modalities are available for the treatment of lunate dislocation, such as proximal row carpectomy, open reduction and internal fixation (ORIF), external fixation, wrist arthrodesis, and lunate excision. In our case, we opted for the lunate excision procedure to avoid anticipated complications, such as arthritis, carpal instability, and risk of pin tract with regard to procedures such as external fixation. Lunate excision in our case led to reduced pain postoperatively and significant improvement in the patient's symptoms with improvement in wrist range of motion and grip strength [8,9].

## Conclusions

Lunate dislocation is a rare injury that occurs in high-energy injuries in adults and may present with CTS. A high index of suspicion, with appropriate clinical and radiological assessments, is important to identify the problem. An accurate diagnosis and treatment of these injuries early is the key to pain relief and the restoration of wrist motion. Early treatment is necessary to prevent adverse complications, such as chronic carpal instability and traumatic arthritis, which are associated with missed or inappropriately treated injuries. The goal is to identify these injuries early and provide early treatment necessary to prevent untoward outcomes that may negatively impact quality of life.
